# Multi-holed fully covered self-expandable metal stent to dilate a benign hepaticojejunostomy anastomotic stricture

**DOI:** 10.1055/a-2717-1802

**Published:** 2025-10-21

**Authors:** Tesshin Ban, Yoshimasa Kubota, Yota Hirayama, Kei Ando, Naoto Imura, Shun Sasoh, Takashi Joh

**Affiliations:** 1 36884 Department of Gastroenterology and Hepatology, Gamagori City Hospital, Gamagori, Japan

In balloon-assisted enteroscopy (BAE)-guided recanalization in patients with benign hepaticojejunostomy anastomotic strictures (HJAS), balloon dilation followed by plastic stent placement maintains long-term recanalization 1 . Temporary placement of a fully covered self-expandable metal stent (FCSEMS) may increase stricture patency rates. However, an FCSEMS may cause acute cholangitis by blocking the biliary bifurcations. Therefore, additional plastic stents are sometimes required to preserve bifurcations 2 3 . A recently developed removable multi-holed FCSMES (MH-FCSEMS) is designed to preserve the biliary branches even when placed unilaterally in the perihilar stricture 4 5 . Herein, we report a patient in whom the HJAS was successfully recanalized without cholangitis using the MH-FCSEMS under BAE guidance.

A 56-year-old female with a history of robotic pancreatoduodenectomy for duodenal papillary cancer presented with fever and gradually worsening serum transaminase levels, which was suspected to be HJAS.

We attempted BAE-guided recanalization of the HJAS using balloon dilation, followed by temporary unilateral placement of the MH-FCSEMS at the anastomosis ( Video 1 ). The HJAS had a pinhole-like appearance on the jejunal side and was 8 mm long ( Fig. 1 **a** , Fig. 2 **a** ). We dilated the HJAS with a 6-mm dilation balloon catheter, followed by deployment of a single MH-FCSEMS (HANAROSTENT Biliary Multi-hole NEO, 10 mm, 5 cm; Boston Scientific, Marlborough, Massachusetts, USA) across the stricture up to the left hepatic duct ( Fig. 2 **b** ). After stent deployment, remnant contrast medium in the bilateral intrahepatic duct was quickly aspirated through the MH-FCSEMS, and these ducts changed into pneumobilia, even though the MH-FCSEMS obstructed the right hepatic duct and segment IV bile duct ( Fig. 2 **c** ). The postprocedural clinical course was uneventful, and the HJAS was successfully recanalized when the stent was removed one month later ( Fig. 1 **b** ).

**Fig. 1:**
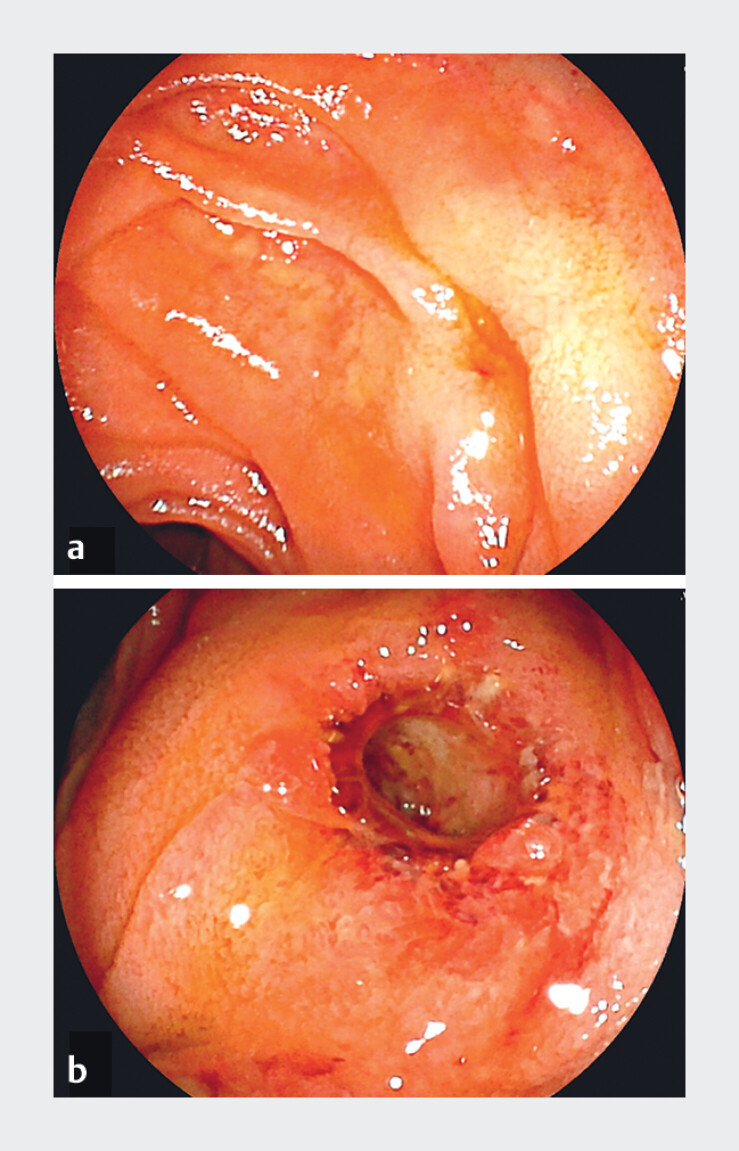
Endoscopic views of hepaticojejunostomy anastomotic stricture. **a** The anastomosis site shows a pinhole-like appearance before placement of a multi-holed fully covered self-expandable metal stent (MH-FCSMES) during balloon-assisted enteroscopy. **b** The anastomosis stricture is considered sufficiently recanalized when the stent is removed after one month from the index procedure.

**Fig. 2:**
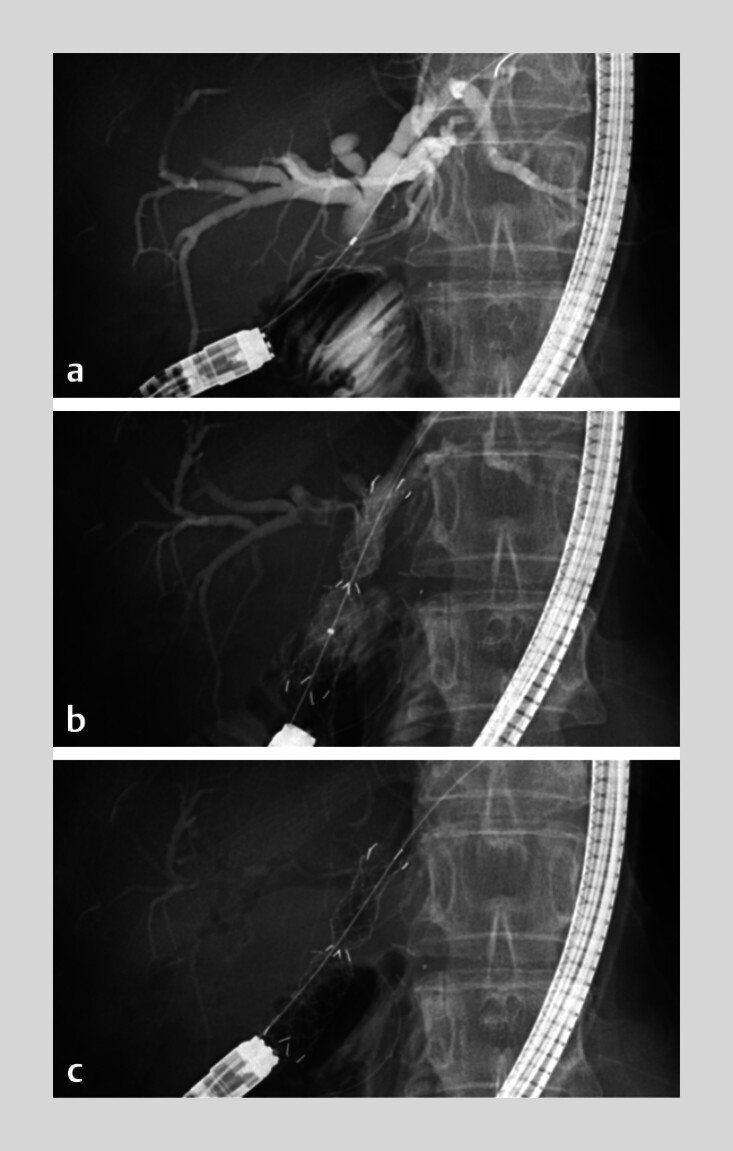
Fluoroscopic images showing the placement of an MH-FCSMES at the hepaticojejunostomy anastomotic stricture during balloon-assisted enteroscopy. **a** The severe stricture measured 8mm in length. **b** The deployed MH-FCSMES is observed to be obstructing the right hepatic duct and the segment IV bile duct. **c** However, multiple holes in the stent membrane allow for the aspiration of bilateral remnant contrast medium and its replacement with the insufflated gas.

Multi-holed fully covered self-expandable metal stent to dilate a benign hepaticojejunostomy anastomotic stricture.Video 1

Temporary placement of the MH-FCSEMS for HJAS is a simple procedure aimed at preventing segmental cholangitis and ensuring adequate stricture dilation.

Endoscopy_UCTN_Code_TTT_1AR_2AZ

## Notes

Endoscopy E-Videos https://eref.thieme.de/e-videos
*E-Videos* is an open access online section of the journal *Endoscopy* , reporting on interesting cases and new techniques in gastroenterological endoscopy. All papers include a high-quality video and are published with a Creative Commons CC-BY license. Endoscopy E-Videos qualify for HINARI discounts and waivers and eligibility is automatically checked during the submission process. We grant 100% waivers to articles whose corresponding authors are based in Group A countries and 50% waivers to those who are based in Group B countries as classified by Research4Life (see: https://www.research4life.org/access/eligibility/ ). This section has its own submission website at https://mc.manuscriptcentral.com/e-videos .
